# Altered Lipid Profile and Oxidative Stress During Pregnancy: Impact on the Fetus and Risk of Metabolic Disorders in Adulthood

**DOI:** 10.3390/ijms27093744

**Published:** 2026-04-23

**Authors:** Kristina Jovanovic, Miljana Z. Jovandaric, Darko Jovanovic, Milos Milincic, Mirjana Krstic, Bojan Cegar, Dimitrije M. Nikolic

**Affiliations:** 1 Department of Neurology, University Children’s Hospital, 11000 Belgrade, Serbia; 2 Department of Neonatology, Clinic for Gynecology and Obstetrics, University Clinical Center of Serbia, 11000 Belgrade, Serbia; 3 Clinic of Urology, University Clinical Center of Serbia, 11000 Belgrade, Serbia; 4 Department of Obstetrics and Gynecology, Clinic for Gynecology and Obstetrics, University Clinical Center of Serbia, 11000 Belgrade, Serbia; 5 Hospital for Gynecology and Obstetrics, Clinical Hospital Center Zemun, 11080 Zemun, Serbia; 6 Faculty of Medicine, University of Belgrade, 11000 Belgrade, Serbia

**Keywords:** pregnancy, lipid metabolism, free fatty acids, oxidative stress, newborn

## Abstract

Pregnancy is characterized by progressive maternal hyperlipidemia, including increased triglycerides, total cholesterol, and low-density lipoprotein, with dynamic fluctuations in high-density lipoprotein. Excess maternal free fatty acids induce oxidative stress through reactive oxygen species, causing mitochondrial dysfunction, lipid peroxidation, activation of inflammatory pathways, and epigenetic remodeling in the placenta and fetal tissues. These molecular alterations impair placental lipid transport and nutrient sensing, leading to hypertrophy of fetal liver, myocardium, and adipose tissue, while disrupting neonatal glucose and lipid homeostasis and increasing susceptibility to perinatal complications and long-term metabolic disorders. This review aims to evaluate mechanistic pathways linking maternal lipid metabolism, oxidative stress, placental function, and fetal organ remodeling. Mechanistic and translational studies were identified through searches of PubMed, Scopus, the Cochrane Library, and Web of Science (2000–2025) using predefined keywords including lipid metabolism, free fatty acids, oxidative stress, placental lipid transport, epigenetics, DNA methylation, fetal programming, and perinatal outcomes. Evidence indicates that maternal lipid imbalance drives placental oxidative and epigenetic modifications, directly contributing to fetal organ hypertrophy and neonatal metabolic dysregulation. In conclusion, maternal dyslipidemia represents a modifiable determinant of fetal organ hypertrophy and long-term metabolic risk, supporting the clinical relevance of maternal lipid monitoring and targeted metabolic interventions during pregnancy.

## 1. Introduction

Pregnancy represents a period of complex physiological and metabolic adaptations in the maternal organism, with the primary goal of providing optimal conditions for fetal growth and development. These adaptations include changes in carbohydrate, lipid, and protein metabolism, hormonal regulation, placental function, oxidative balance, and epigenetic modifications, which together shape the intrauterine environment. Understanding these changes is crucial for identifying risk factors that may lead to adverse perinatal outcomes, as well as long-term metabolic and cardiometabolic disorders in adulthood [1].

During pregnancy, significant changes occur in carbohydrate metabolism. Insulin resistance increases, particularly in the second and third trimesters, allowing for the maintenance of elevated circulating glucose levels to ensure a constant energy supply for the fetus. The maternal liver and placenta contribute to gluconeogenesis, while the fetal liver gradually acquires the capacity for glycogen storage and glycemic regulation. Increased glucose availability supports not only energy demands for growth but also fetal lipid synthesis, which is particularly important for brain and lung development [2].

Alterations in lipid metabolism during pregnancy are profound. Concentrations of triglycerides, total cholesterol, and low-density (LDL) increase, while the levels of high-density lipoprotein (HDL) vary throughout gestation, often showing a mild decline during the second trimester and stabilization in the third trimester. These changes facilitate lipid transport to the fetus, which is required for the synthesis of membrane phospholipids, steroid hormones, and the accumulation of energy reserves. At the same time, elevated levels of free fatty acids (FFAs) in the maternal circulation contribute to the generation of reactive oxygen species (ROS), leading to oxidative stress that may cause mitochondrial dysfunction and lipid peroxidation [3].

Hormonal signals of pregnancy play a central role in these processes. Estrogen stimulates hepatic lipoprotein synthesis, increasing levels of LDL and total cholesterol, while progesterone modulates lipolysis in adipose tissue, promoting the release of FFAs. Human placental lactogen (hPL) induces physiological insulin resistance, thereby increasing the availability of glucose and lipids for the fetus. In addition, cortisol, thyroid hormones, and leptin contribute to the fine regulation of maternal metabolism and nutrient allocation according to fetal demands [4].

This association was further confirmed in a study conducted by Jovandaric MZ and Ivanovski PI, which demonstrated significantly lower circulating free fatty acid levels in newborns of mothers with gestational diabetes mellitus compared with healthy controls, together with differences in neonatal anthropometric parameters, including higher birth weight in the gestational diabetes group; the authors interpreted these findings in the context of altered fetal lipid metabolism, characterized by increased insulin activity and suppression of lipolysis, which may promote enhanced lipid storage and contribute to increased fetal growth [5].

The placenta is a central regulator of nutrient and lipid transfer. Maternal triglycerides are hydrolyzed into FFAs and glycerol by lipoprotein lipases, and non-essential fatty acids can be synthesized from carbohydrates and acetate in fetal tissues. These substrates are used for membrane phospholipid synthesis, steroid hormone production, and energy storage. Oxidative stress alters the expression of enzymes and transport proteins in the placenta, increasing the transfer of FFAs and lipid peroxides to the fetus. Increased exposure to oxidative stress may contribute to fetal tissue hypertrophy, altered lipid profiles, and organ dysfunction [6].

Epigenetic changes associated with altered lipid metabolism and oxidative stress include DNA methylation of genes involved in metabolic regulation, histone modifications, and microRNA-mediated regulation, which influence the expression of genes responsible for lipid homeostasis and insulin signaling pathways. These mechanisms reflect the capacity of metabolic and redox signals to modulate epigenetic regulatory networks, thereby affecting key processes involved in energy metabolism and insulin sensitivity. These changes contribute to fetal programming, a long-term adaptive process in response to the intrauterine environment, with potential consequences such as persistent alterations in metabolic regulation and an increased risk of metabolic and cardiometabolic disorders later in life, including insulin resistance, dyslipidemia, hypertension, and cardiovascular diseases [7].

Saturated fatty acids induce a proinflammatory response in human trophoblastic cells through activation of Toll-like receptor 4-dependent signaling pathways. This activation triggers downstream NF-κB signaling, resulting in increased expression and secretion of proinflammatory cytokines, including IL-6 and TNF-α. These findings indicate a direct mechanistic link between lipid exposure and innate immune activation at the maternal–fetal interface, highlighting the capacity of placental trophoblasts to respond to metabolic stimuli through classical inflammatory pathways [8].

The fetal organism responds to these changes through adaptive modifications in the liver, pancreas, cardiovascular system, and adipocytes. The fetal liver increases lipogenesis and triglyceride storage, the pancreas enhances insulin secretion, the heart exhibits hypertrophic changes, and adipocytes accumulate lipids. Collectively, these adaptations contribute to increased neonatal body mass and may predispose the offspring to postnatal dyslipidemia, obesity, and insulin resistance [9].

All these metabolic alterations—including gestational hypertriglyceridemia, elevated low-density lipoprotein concentrations, dynamic changes in high-density lipoproteins, increased circulating free fatty acids, progressive insulin resistance, enhanced proteolysis, redox imbalance, and activation of inflammatory signaling pathways—collectively modulate the intrauterine milieu and influence fetal metabolic adaptation. Detailed characterization of these interconnected processes facilitates risk stratification and supports the development of targeted preventive strategies, including evidence-based nutritional modulation and redox-regulatory interventions, to improve perinatal outcomes and minimize early metabolic vulnerability in the offspring [10].

Although maternal lipid metabolism and fetal programming have been extensively reviewed, existing literature has largely treated gestational dyslipidemia as a descriptive physiological phenomenon or focused on clinical outcomes without integrating specific maternal lipid fractions, placental lipid transport, redox-sensitive inflammatory signaling, and epigenetic regulation into a unified mechanistic framework [11]. Previous reviews have summarized maternal lipid profile changes and their associations with maternal and neonatal outcomes, and systematic lipidomic analyses have identified associations between lipid signatures and pregnancy complications; however, a comprehensive synthesis linking these lipid alterations to the molecular mechanisms of placental oxidative stress and fetal metabolic programming remains insufficiently defined. Similarly, recent work on placental inflammation and oxidative stress in the context of maternal obesity highlights key pathways. Nevertheless, it does not fully integrate lipid-specific signaling with epigenetic fetal adaptation processes. This review addresses this gap by proposing an integrated maternal–placental–fetal framework that connects gestational lipid remodeling with oxidative, inflammatory, and epigenetic pathways governing developmental programming [12].

## 2. Literature Search Strategy and Study Organization

### 2.1. Search Strategy

A systematic and comprehensive search of the literature was performed to identify relevant studies investigating the effect of maternal lipid profiles and oxidative stress on fetal development and long-term metabolic programming. The search was carried out across major electronic databases, including PubMed/MEDLINE, Scopus, and Web of Science, covering the period from 2000 to 2025. The search strategy employed a combination of keywords and Medical Subject Headings (MeSH) terms to ensure broad coverage: “pregnancy,” “lipid metabolism,” “free fatty acids,” “oxidative stress,” “placental lipid transport,” “epigenetics,” “DNA methylation,” and “fetal programming.” Only peer-reviewed articles published in English were included. We focused on original research, meta-analyses, and high-impact reviews that offered mechanistic insights into the maternal–fetal metabolic axis. Among the identified records, selections were made based on their scientific rigor, relevance to molecular signaling, and contribution to the integrative framework discussed in this review.

### 2.2. Study Organization

The present review is structured into thematic sections to provide a coherent overview of the molecular and clinical aspects of maternal–fetal metabolism. Following this methodological section, this paper is organized as follows: Section 3 explores the physiological adaptations of maternal lipid metabolism. Section 4 examines the role of free fatty acids (FFAs) and the generation of oxidative stress. Section 5 focuses on the mechanisms of placental lipid transport and oxidative modifications. Section 6 discusses fetal exposure to dyslipidemia and inflammatory crosstalk, including neurodevelopmental implications. Section 7 provides an in-depth analysis of the epigenetic mechanisms linking maternal lipids to fetal programming. Finally, Section 8 offers integrative strategies for maternal metabolic optimization, followed by concluding remarks.

## 3. Physiological Adaptations of Lipid Metabolism During Pregnancy

Pregnancy represents a period of complex metabolic and physiological adaptations in the maternal organism, with the primary goal of providing optimal conditions for fetal growth and development. Changes in lipid metabolism constitute one of the most significant adaptations, as they ensure adequate energy and structural substrate supply to the fetus while simultaneously supporting the mother’s energetic demands throughout gestation [13].

Alterations in lipid profiles during pregnancy are gestational-phase-dependent. In the first trimester, a mild increase in lipid synthesis occurs, while in the second and third trimesters, there is a marked rise in triglycerides, total cholesterol, and low-density lipoproteins (LDL). These changes facilitate lipid transport to the fetus, which is essential for membrane phospholipid synthesis, steroid hormone production, and energy reserve accumulation. High-density lipoprotein (HDL) shows variable changes, often slightly decreasing in mid-gestation and stabilizing before delivery [14].

Placental lipid metabolism involves the conversion of circulating maternal lipids into free fatty acids (FFAs), which are subsequently used for fetal growth and development. The placenta actively metabolizes lipids via lipoprotein lipases and transport proteins, and FFA transport across the placental barrier is highly regulated, providing the fetus with a constant supply of essential fatty acids, particularly long-chain polyunsaturated fatty acids needed for brain and retinal development [15].

Free fatty acids released from maternal adipose tissue via lipolysis increase during pregnancy due to progressive insulin resistance and hormonal influences, including human placental lactogen, estrogen, and progesterone. This metabolic adaptation enhances lipid substrate availability for maternal and fetal energy demands. Elevated circulating free fatty acids reflect increased adipose tissue lipolysis and contribute to the characteristic shift toward greater lipid utilization in late gestation [16].

Hormonal regulation is central to metabolic adaptations during pregnancy. Estrogen contributes to increased hepatic lipoprotein synthesis and rising circulating lipid levels, including low-density lipoprotein and total cholesterol. Progesterone and human placental lactogen promote maternal lipolysis and physiological insulin resistance, thereby increasing the availability of glucose and lipid substrates for fetal and placental energy demands. Additional endocrine factors, including cortisol, thyroid hormones, and leptin, contribute to the fine regulation of maternal metabolism and nutrient partitioning in accordance with fetal requirements [17].

Protein metabolism also adapts during pregnancy through increased proteolysis in skeletal muscle and other tissues, releasing amino acids that serve as substrates for fetal protein synthesis and placental/fetal gluconeogenesis. These dynamic changes in maternal plasma amino acid profiles are closely linked to fetal growth and neonatal anthropometric outcomes, reflecting a coordinated adaptation of maternal energy resources throughout gestation [18].

The placenta serves as the primary regulator of lipid transport between the mother and fetus. Lipoprotein lipases hydrolyze circulating triglycerides into FFAs and glycerol, which are then transferred via specific transport proteins (e.g., FATP, CD36, FABP) into trophoblast cells and subsequently into fetal circulation. This process is dynamic and adapts to fetal growth requirements, particularly during the third trimester, when lipid supply is crucial for neurodevelopment and energy reserve accumulation [19].

**Figure 1:**
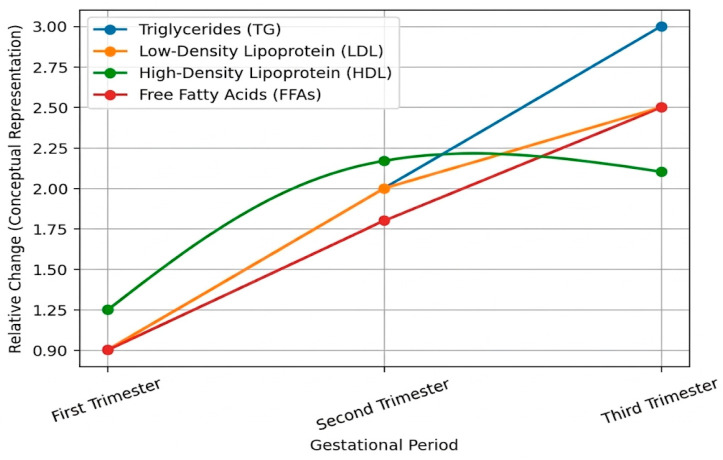
Trimester-specific changes in maternal lipid fraction.

These adaptations represent a sophisticated regulatory network that enables optimal energy and substrate transport to the fetus but may also establish the basis for potential risks. ROS accumulation due to intensified lipid metabolism and imbalanced antioxidant defenses may contribute to oxidative stress and increase the risk of pregnancy complications, such as preeclampsia, intrauterine growth restriction, and metabolic disorders in the offspring later in life [20] (Figure 1).

During the first trimester, pregnancy is characterized by a predominantly anabolic metabolic state aimed at establishing maternal energy reserves that will support the increasing demands of later gestation. Lipid metabolism during this period shows only modest alterations. Total cholesterol and low-density lipoprotein (LDL) concentrations begin to rise slightly, largely under the stimulatory influence of estrogen on hepatic lipoprotein synthesis. High-density lipoprotein (HDL) levels are generally maintained or may show a mild increase, while triglyceride concentrations remain close to preconception values. Insulin sensitivity is relatively preserved, favoring lipogenesis and lipid storage within maternal adipose tissue. This early accumulation of lipid stores represents a preparatory mechanism that ensures sufficient substrate availability for the rapid fetal growth that occurs in subsequent trimesters.

In the second trimester, maternal metabolism gradually shifts from an anabolic to a more catabolic profile. Triglyceride levels increase more markedly, accompanied by further elevations in total cholesterol and LDL. HDL concentrations may begin to decline slightly during mid-gestation. Hormonal factors, particularly human placental lactogen, progesterone, and estrogen, promote lipolysis in maternal adipose tissue and contribute to the development of physiological insulin resistance. As a result, circulating free fatty acid concentrations increase. This transition enhances the mobilization of lipid substrates and supports increased placental transfer of fatty acids, including long-chain polyunsaturated fatty acids essential for fetal neurodevelopment and membrane biosynthesis. The second trimester, therefore, represents a dynamic metabolic adjustment phase in which nutrient allocation progressively prioritizes fetal requirements.

The third trimester is characterized by a pronounced catabolic state and represents the period of maximal metabolic demand. Maternal triglyceride concentrations typically reach their highest levels, often two- to threefold above pre-pregnancy values, while LDL remains significantly elevated. HDL levels generally stabilize or remain slightly reduced compared to early pregnancy. Circulating free fatty acids are substantially increased due to intensified lipolysis and marked insulin resistance. Placental lipoprotein lipase activity is enhanced, facilitating the hydrolysis of maternal triglycerides and the transfer of free fatty acids into the fetal circulation. This intensified lipid mobilization ensures adequate substrate supply for rapid fetal weight gain, adipose tissue deposition, brain maturation, retinal development, and surfactant synthesis in the lungs. However, the heightened metabolic flux is also associated with increased mitochondrial activity and reactive oxygen species production, which may contribute to oxidative stress if antioxidant defenses are insufficient. Collectively, these trimester-specific adaptations reflect a finely regulated physiological process that optimizes fetal growth while maintaining maternal metabolic balance.

## 4. Free Fatty Acids and Oxidative Stress in Pregnancy

Free fatty acids (FFAs) in maternal circulation are mostly bound to serum albumin (SA). At the same time, a small proportion of unbound FFAs allows for active cellular uptake and passive transport across the placenta. Transport of FFAs across the syncytiotrophoblast membrane is mediated by specific transporter proteins, including CD36 (fatty acid translocase) and fatty acid transport proteins (FATPs), enabling the uptake of long-chain fatty acids into trophoblast cells and their distribution to the fetus [21].

After cellular entry, FFAs are activated into acyl-coenzyme A (acyl-CoA) derivatives by acyl-CoA synthetases, making them substrates for mitochondrial β-oxidation, re-esterification, or metabolic signaling pathways that regulate energy metabolism and gene expression. Mitochondrial transport of long-chain acyl-CoA esters depends on the carnitine transport system, including carnitine palmitoyltransferase 1 (CPT1), carnitine-acylcarnitine translocase (CACT), and carnitine palmitoyltransferase 2 (CPT2), as these molecules cannot directly cross the inner mitochondrial membrane [22].

Increased flux of FFAs into placental mitochondria raises the production of reducing agents, specifically nicotinamide adenine dinucleotide in its reduced form (NADH) and flavin adenine dinucleotide in its reduced form (FADH_2_), which donate electrons to the electron transport chain (ETC) [23]. This increased electron input can temporarily overload the ETC, causing electrons to leak to molecular oxygen and form reactive oxygen species (ROS) [24]. In normal pregnancy, these ROS are produced naturally as part of increased placental metabolic and mitochondrial activity, contributing to redox signaling that supports trophoblast differentiation, angiogenesis, and overall placental adaptation without indicating harmful oxidative stress [25]. ROS, such as superoxide anion (O_2_•^−^) and hydrogen peroxide (H_2_O_2_), are generated during cellular metabolism and oxidative stress, and when their levels surpass the cell’s antioxidant defenses, lipid peroxidation (LPO) occurs, creating reactive aldehydes like malondialdehyde (MDA) and 4-hydroxy-2-nonenal (4-HNE), which are primary products of lipid peroxidation and are involved in damaging membranes and cellular components [26,27]. In the human placenta, oxidative stress directly impairs fatty acid oxidation and ATP synthesis, indicating compromised β-oxidation and energy production [28].

Placental accumulation of FFAs and ROS activates redox-sensitive signaling pathways, including nuclear factor kappa B (NF-κB) and other transcription factors associated with inflammation and antioxidant responses. Activation of these pathways increases the expression of proinflammatory cytokines and modulates fatty acid transporter expression, while induction of antioxidant mechanisms, including superoxide dismutase (SOD), catalase (CAT), and glutathione peroxidase (GPx), provides adaptive protection against ROS [29,30].

Chronic exposure to elevated FFAs and oxidative signals can impair trophoblast function, induce proinflammatory states, and reduce placental metabolic efficiency, which is associated with pregnancy complications, including preeclampsia and fetal growth restriction [31]. Metabolic stress caused by high FFA and ROS concentrations can transmit metabolic imbalance to the fetus, affecting mitochondrial function and the expression of antioxidant enzymes, thereby increasing the postnatal risk of metabolic disorders, including insulin resistance and dyslipidemia [32].

## 5. Placental Lipid Transport and Oxidative Modifications

Placental lipid transport is a tightly regulated, multistep process that delivers maternal free fatty acids (FFAs) to the developing fetus, supporting growth, differentiation, and organogenesis. Maternal FFAs are liberated from circulating triglyceride-rich lipoproteins via lipoprotein lipase (LPL) and endothelial lipase (EL) at the maternal-facing microvillous membrane of the syncytiotrophoblast, generating FFAs and monoacylglycerols (MAGs) available for cellular uptake. LPL primarily hydrolyzes triglycerides to release FFAs, whereas EL also liberates phospholipid-derived fatty acids, enriching the pool of long-chain polyunsaturated fatty acids (LC-PUFAs) for placental uptake [33].

These FFAs are subsequently translocated across trophoblast membranes by specialized transporter proteins, including fatty acid translocase (CD36), various fatty acid transport protein (FATP) isoforms, and cytoplasmic fatty acid-binding proteins (FABPs). CD36 facilitates the uptake of long-chain fatty acids, particularly DHA and AA, for fetal neurodevelopment. FATP isoforms (e.g., FATP1, FATP4) possess acyl-CoA synthetase activity, enabling intracellular activation of FFAs and directing them toward β-oxidation, triglyceride esterification, phospholipid synthesis, or storage. FABPs act as intracellular chaperones, binding FFAs and directing them to mitochondria or lipid droplets according to cellular energy requirements and redox state [34].

The expression and localization of these transporters are dynamically regulated by nuclear receptors, notably peroxisome proliferator-activated receptors (PPARs) and liver X receptors (LXRs), which integrate maternal lipid availability with placental metabolic programming. PPARs regulate transcription of CD36, FATPs, and FABPs, modulating both uptake and intracellular trafficking of FFAs, while LXRs respond to cholesterol derivatives and influence FATP expression and membrane localization [35].

Long-chain polyunsaturated fatty acids (LC-PUFAs), including DHA and AA, are selectively enriched in fetal circulation through transporter affinity and intracellular handling, providing critical substrates for neurodevelopment. The activity of CD36 and FATPs ensures preferential transport of these essential fatty acids, while FABPs coordinate intracellular partitioning to meet fetal metabolic demands [36].

Oxidative stress, defined as an imbalance between reactive oxygen species (ROS) production and antioxidant defenses, modulates placental lipid handling at multiple levels. Excessive ROS, arising from mitochondrial respiration, inflammatory signaling, or metabolic overload, induces lipid peroxidation of trophoblast membranes, producing reactive aldehydes such as malondialdehyde (MDA), which compromise membrane fluidity and transporter function [37]. CD36 and FATPs are particularly susceptible to oxidative modifications (carbonylation, nitrosylation, altered membrane trafficking), reducing FFA uptake efficiency and impairing downstream intracellular processing [36]. ROS-mediated mitochondrial damage further decreases β-oxidation capacity, reduces ATP production, and leads to the accumulation of lipid intermediates [38].

Beyond direct oxidative modifications, ROS influence intracellular signaling pathways. Redox-sensitive cascades, including nuclear factor kappa B (NF-κB) and mechanistic target of rapamycin complex 1 (mTORC1), modulate the expression and activity of lipid transporters and FABPs, linking oxidative stress to altered nutrient allocation and inflammatory responses [38]. Chronic oxidative stress and dysregulated lipid transport are consistently observed in pregnancies complicated by preeclampsia, gestational diabetes mellitus, and intrauterine growth restriction. These conditions exhibit increased placental lipid peroxidation, altered antioxidant enzyme activity, and impaired fetal nutrient delivery [39].

**Figure 2:**
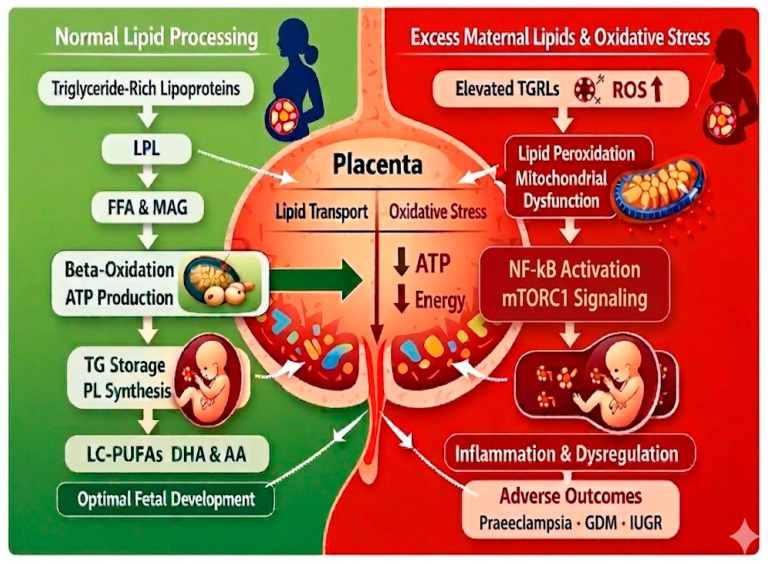
Molecular mechanisms of placental lipid transport and oxidative-stress-induced pathophysiological cascades.

Collectively, these mechanistic insights demonstrate that placental lipid transport and oxidative modifications constitute an interconnected molecular and cellular network shaping fetal metabolic programming, with implications for long-term health outcomes and susceptibility to metabolic disorders in later life. The selective vulnerability of CD36, FATPs, and FABPs to oxidative stress highlights their central role in coordinating maternal–fetal lipid transfer and underscores the impact of redox imbalance on fetal organ development [40] (Figure 2).

The illustration depicts the metabolic pathways of lipid processing at the fetoplacental unit, highlighting the distinction between physiological homeostatic processes and pathological modifications. Under physiological conditions, maternal triglyceride-rich lipoproteins (TGRLs) undergo extracellular hydrolysis mediated by the enzyme lipoprotein lipase (LPL), releasing free fatty acids (FFAs) and monoglycerides (MAGs) for placental uptake. These substrates enter mitochondrial beta-oxidation, ensuring high adenosine triphosphate (ATP) production necessary for maintaining placental energy homeostasis. Subsequent intracellular pathways involve the storage of triglycerides (TGs) and phospholipid (PL) synthesis, with a prioritized transfer of long-chain polyunsaturated fatty acids (LC-PUFAs), primarily docosahexaenoic acid (DHA) and arachidonic acid (AA), which are essential for optimal fetal development. Conversely, excess maternal lipids induce elevated reactive oxygen species (ROS) production, initiating lipid peroxidation and progressive mitochondrial dysfunction. This energetic collapse, characterized by a significant depletion of ATP levels, activates pro-inflammatory signaling pathways, predominantly nuclear factor-kappa B (NF-κB) and mammalian target of rapamycin complex 1 (mTORC1) signaling. Chronic inflammation and the resulting dysregulation of transport mechanisms directly converge toward adverse perinatal outcomes, including preeclampsia, gestational diabetes mellitus (GDM), and intrauterine growth restriction (IUGR).

## 6. Fetal Exposure to Dyslipidemia, Oxidative Stress, and Inflammatory Crosstalk

Fetal exposure to maternal dyslipidemia creates a biochemical milieu characterized by excessive ROS generation, cytokine activation, and disrupted redox homeostasis [41]. Under physiological conditions, ROS and reactive nitrogen species (RNS) act as essential signaling molecules regulating cellular differentiation, organogenesis, and metabolic maturation. However, maternal lipid imbalance drives chronic ROS accumulation, surpassing fetal antioxidant capacity and directly interfering with organ development, lipid metabolism, and energy homeostasis. This environment promotes activation of redox-sensitive transcription factors, particularly nuclear factor kappa B (NF-κB), which mediates transcription of pro-inflammatory cytokines including tumor necrosis factor-alpha (TNF-α), interleukin-6 (IL-6), and interleukin-1 beta (IL-1β), establishing a feed-forward loop between oxidative stress and inflammation [42].

Placental transfer of oxidized lipids, lipid peroxidation products, and inflammatory mediators allows oxidative and inflammatory signals to propagate into fetal circulation, affecting multiple organs simultaneously [43]. Within the fetal compartment, ROS disrupt mitochondrial β-oxidation by inducing structural and functional damage to electron transport chain complexes, reducing adenosine triphosphate (ATP) production, and favoring accumulation of partially oxidized lipid intermediates [44]. These mechanisms contribute to early metabolic inflexibility, ectopic lipid deposition, and impaired glucose handling, predisposing the neonate to insulin resistance and dyslipidemia [45]. Experimental models demonstrate that ROS-driven NF-κB activation in hepatocytes and pancreatic progenitor cells mediates oxidative damage and cytokine release, amplifying postnatal metabolic dysregulation [46].

The fetal liver is particularly vulnerable to combined oxidative and inflammatory stress due to its central role in lipid and glucose metabolism [47]. Intrauterine ROS exposure impairs fatty acid catabolism and reduces antioxidant gene expression. Maternal malnutrition induces ROS accumulation and dysregulated hepatic lipid metabolism, supporting a mechanistic link between redox imbalance and impaired fetal lipid homeostasis [48]. Similarly, maternal protein deficiency reduces peroxisome biogenesis, increases ROS, and dysregulates fatty acid metabolism enzymes in fetal liver, contributing to oxidative damage and potential lipid accumulation [49]. Chronic high-fat maternal diet increases triglyceride deposition in fetal liver tissue, consistent with early dyslipidemic changes associated with ROS [50].

Concomitant NF-κB-driven cytokine signaling inhibits insulin receptor pathways, disrupts gluconeogenesis, and enhances susceptibility to postnatal insulin resistance [51]. Pancreatic development is highly sensitive to oxidative-inflammatory crosstalk: excessive ROS and pro-inflammatory cytokines compromise endocrine progenitor cell survival, reduce β-cell mass, and impair insulin secretory function via mitochondrial dysfunction and oxidative DNA damage, setting the stage for early-life dysglycemia [52].

Cardiac tissue undergoes fetal programming in response to dyslipidemia-induced oxidative and inflammatory stress. ROS-mediated mitochondrial dysfunction and NF-κB-dependent signaling disrupt cardiomyocyte calcium handling, sarcomeric organization, and energy metabolism, triggering compensatory hypertrophy and increasing long-term cardiovascular vulnerability [53]. Adipose tissue development is similarly affected: intrauterine oxidative stress and cytokine signaling modify adipocyte differentiation via redox-sensitive transcription factors, favoring lipid storage, pro-inflammatory phenotype, and reduced insulin sensitivity, contributing to disproportionate neonatal adiposity [54].

At the molecular level, ROS and inflammation converge to influence epigenetic regulation and transcriptional networks. Persistent ROS activate mitogen-activated protein kinase (MAPK) and c-Jun N-terminal kinase (JNK) pathways, while cytokines engage NF-κB cascades. These pathways modulate genes controlling lipid metabolism, glucose homeostasis, and energy utilization [55]. Lipid peroxidation products, including malondialdehyde and 4-hydroxynonenal, modify proteins and nucleic acids, amplifying oxidative and inflammatory damage [56]. Mitochondrial DNA is highly susceptible to oxidative injury, impairing respiratory chain function and promoting further ROS overproduction. Nuclear lipid sensors, including peroxisome proliferator-activated receptors alpha and gamma (PPARα, PPARγ) and sterol regulatory element-binding protein-1c (SREBP-1c), become dysregulated, perturbing lipid oxidation and lipogenesis balance [57].

Vascular development is impaired: ROS and cytokines reduce nitric oxide bioavailability, impair endothelial function, and promote vasoconstriction, compromising tissue perfusion and organ-specific metabolic stress [58]. Chronic oxidative-inflammatory signaling also disrupts autophagy–apoptosis balance, leading to premature cellular senescence or apoptosis in metabolically active fetal tissues, further increasing susceptibility to postnatal metabolic disease [59]. Within this intrauterine context, these interconnected mechanisms establish a fetal metabolic phenotype prone to cardiometabolic dysfunction, adipose inflammation, insulin resistance, and organ-specific remodeling, which persists postnatally [60].

Altered lipid profiles modulate the structural and signaling dynamics of neuronal membranes. The incorporation of polyunsaturated fatty acids (PUFAs), especially docosahexaenoic acid (DHA) and arachidonic acid (AA), into phospholipids contributes to membrane fluidity, which is crucial for the function of receptors and ion channels (NMDA, AMPA, GABA-A) and the interaction of synaptic proteins [61]. Dyslipidemia or hyperlipidemia can reduce the placental transport of DHA and AA due to the downregulation of lipoprotein lipases (LPL) and transport proteins (FABP, FATP), thereby limiting fetal availability [62].

DHA improves the formation of lipid rafts, organizes signaling molecules and facilitates the activation of kinases (PKC, CaMKII), regulating the phosphorylation of synaptic proteins and synaptogenesis [63]. Placental transfer of DHA requires Mfsd2a and the lipoprotein receptors LDLR and LRP1; they can be reduced in gestational diabetes and preeclampsia, mediated by oxidative and inflammatory pathways [64]. DHA modulates Wnt/b-catenin and Notch signaling, and cytoskeletal reorganization via Rho GTPases, promoting proper neuronal migration and cortical layer formation. DHA deficiency reduces Nestin and doublecortin (DCX) expression, impairing neurogenesis and synaptogenesis [65].

DHA affects the metabolism of glutamate and GABA through the regulation of glutamine synthetase and glutamate decarboxylase, and serotonergic signaling through the serotonin transporter (SERT). PUFA metabolites act as lipid mediators by activating the Src-family of kinases via phosphatidylserine-rich lipid rafts, modulating MAPK cascades [66]. Epigenetic effects of lipid metabolites, including DNA methylation, histone modifications, and regulation of non-coding RNA, contribute to long-lasting changes in neurodevelopmental gene expression. An imbalance of n-3/n-6 PUFA increases pro-inflammatory eicosanoids (PGE2, LTB4) and activates NF-κB, enhancing oxidative stress in the placenta and fetal brain [67].

Epidemiological evidence links adequate maternal DHA with favorable neurocognitive, visuomotor, and socio-emotional trajectories in early childhood, while low DHA increases the risk of attention deficits and cognitive impairments, mediated by combined biochemical and epigenetic mechanisms [68].

Emerging clinical evidence from prospective cohort and longitudinal studies indicates that maternal dyslipidemia during pregnancy is associated with measurable neonatal and childhood metabolic outcomes. Elevated maternal triglycerides, total cholesterol, and LDL cholesterol in early pregnancy have been linked to higher birth weight and an increased risk of large-for-gestational-age (LGA) and macrosomic births, independent of maternal BMI and glycemic control, suggesting that supraphysiological lipid exposure in utero contributes to fetal overgrowth [69]. Maternal lipid concentrations measured in early pregnancy also correlate with offspring lipid profiles at ages 6 and 10 years, indicating a long-term influence on cardiovascular risk biomarkers beyond birth [70]. Community-based cohort analyses found that higher maternal triglycerides and LDL cholesterol in early gestation were associated with greater adiposity markers, including BMI, waist-to-height ratio, and fat percentage, in preadolescence, highlighting a potential role of the prenatal lipid environment in shaping postnatal body composition [71]. Other clinical studies show that maternal and cord blood triglycerides are elevated in pregnancies complicated by obesity and correlate positively with neonatal birth and placental weights, predisposing offspring to later dyslipidemia and insulin resistance. Maternal dyslipidemia, particularly in pregnancies complicated by obesity, is associated with elevated maternal and cord blood triglyceride levels, which correlate positively with neonatal birth weight and placental weight, predisposing offspring to later dyslipidemia and insulin resistance [72]. In a prospective cohort study by Beneventi et al., obese pregnant women exhibited significantly higher concentrations of total cholesterol and triglycerides compared with normoweight controls, and cord blood triglyceride levels were similarly elevated, reflecting increased fetal exposure to lipids. Both maternal and cord blood triglycerides, as well as the TG/HDL-C ratio, were positively associated with neonatal birth weight percentiles and placental mass, with infants born large for gestational age demonstrating higher triglyceride levels and TG/HDL-C ratios than those who were not LGA. These findings indicate that maternal and fetal lipid excess contribute to enhanced fetal growth and placental development, and may program early metabolic susceptibility in the offspring [73]. Furthermore, data from the Amsterdam Born Children and their Development (ABCD) study demonstrate associations between maternal early pregnancy lipid profiles and childhood lipid levels and adiposity markers at school age, independent of maternal diet and adiposity, reinforcing the concept that intrauterine lipid exposure has lasting metabolic consequences. Collectively, these findings from multi-center cohorts and longitudinal follow-ups support that maternal lipid homeostasis—independent of glucose status—plays a significant role in fetal metabolic programming, offspring adiposity, and long-term cardiometabolic risk, with implications for early prenatal screening and interventions [74].

Male and female fetuses exhibit distinct responses to maternal metabolic stressors, including dyslipidemia and oxidative stress, due to sex-dependent placental adaptations. Clinical and experimental data indicate that male placentas demonstrate altered regulation of fatty acid metabolism, nutrient transport, and increased oxidative stress markers, whereas female placentas show compensatory mechanisms such as enhanced mitochondrial biogenesis and lipid synthesis, suggesting differential vulnerability and adaptive capacity [75,76]. Transcriptomic analyses further reveal sexually dimorphic gene expression patterns in placentas exposed to gestational metabolic disorders, including differential regulation of glycemic and inflammatory pathway genes in conditions such as gestational diabetes, emphasizing the role of fetal sex in placental metabolic programming [77]. Epigenetic studies corroborate these findings, showing sex-specific DNA methylation patterns and differential expression of imprinted genes, which can influence growth trajectories and long-term cardiometabolic risk in offspring [78].

## 7. Epigenetic Mechanisms Linking Maternal Lipid Profile to Fetal Programming

Pregnancy involves a steady increase in circulating lipids, which serve not only as energy sources but also as signaling molecules capable of influencing gene regulation through DNA methylation and other epigenetic mechanisms. Early-pregnancy maternal dyslipidemia has been linked to altered DNA methylation at placental sites relevant to lipid metabolism and energy balance, indicating that maternal lipid levels can shape the epigenetic landscape of the placenta [79]. Additionally, maternal triglyceride levels are related to different DNA methylation patterns in cord blood at genes involved in lipid metabolism, and these methylation patterns are connected to measures of child adiposity, suggesting that maternal lipid exposure might program offspring metabolic traits that last into postnatal life [80].

Epigenetic regulation encompasses a set of molecular mechanisms that modulate gene expression without altering the underlying DNA sequence, primarily through DNA methylation, post-translational histone modifications, and non-coding RNA-mediated regulation. These mechanisms are particularly active during embryonic and fetal development, when rapid cell proliferation and differentiation require precise control of transcriptional programs [81]. The placenta, as a highly metabolically active and epigenetically dynamic organ, represents a critical interface through which maternal metabolic signals, including lipid-derived cues, are transmitted to the fetus. Variations in maternal lipid profiles have been shown to influence placental epigenetic patterns, thereby modulating placental transport capacity, mitochondrial function, inflammatory signaling, and antioxidant defenses [82].

DNA methylation is the most extensively studied epigenetic mechanism linking maternal lipid metabolism to fetal programming in humans. This process involves the addition of methyl groups to cytosine residues at CpG dinucleotides, leading to changes in chromatin structure and transcriptional activity. Recent human studies have demonstrated that maternal circulating lipid levels are associated with differential DNA methylation at loci involved in lipid transport, fatty acid metabolism, and energy homeostasis in both placental tissue and cord blood. In particular, genes encoding lipoprotein lipase (LPL), fatty acid transporters, and regulators of lipid sensing have been identified as epigenetically responsive to maternal lipid status, suggesting that maternal lipid availability directly shapes the epigenetic regulation of placental lipid handling [83].

Placental DNA methylation patterns seem to reflect not only total lipid levels but also qualitative features of maternal lipid profiles, such as the ratio between saturated and unsaturated fatty acids. Epigenome-wide association studies have identified specific differentially methylated regions linked to maternal triglyceride and cholesterol levels, many of which are associated with genes involved in mitochondrial metabolism, oxidative phosphorylation, and insulin signaling. These results suggest that maternal lipid exposure during pregnancy can cause coordinated epigenetic changes in metabolic pathways that are vital for placental energy production and nutrient transfer. Importantly, such methylation changes have been observed in uncomplicated pregnancies, supporting the notion that normal variations in maternal lipid metabolism can still influence differences in fetal programming [84].

Cord blood-based analyses further support a role for maternal lipids in shaping fetal epigenetic landscapes. Associations between maternal triglyceride concentrations and DNA methylation at CpG sites within genes related to lipid metabolism, immune regulation, and mitochondrial function have been reported, and some of these epigenetic marks have been linked to measures of adiposity and metabolic risk in early childhood. These observations suggest that lipid-associated epigenetic signatures established in utero may persist beyond birth and influence postnatal metabolic phenotypes. The persistence of such marks underscores their potential role as mediators of developmental programming rather than transient adaptations to the intrauterine environment [85].

In addition to DNA methylation, histone modifications constitute a dynamic and metabolically sensitive layer of epigenetic regulation that links maternal lipid metabolism to the expression of genes in the placenta and fetus. Histone acetylation, methylation, and other post-translational modifications influence chromatin accessibility and transcriptional activity and are directly coupled to cellular metabolic states. Key metabolites derived from lipid metabolism, including acetyl-CoA and NAD^+^, serve as substrates or cofactors for chromatin-modifying enzymes such as histone acetyltransferases, histone deacetylases, and sirtuins. Consequently, changes in placental lipid oxidation and mitochondrial activity induced by maternal lipid availability can alter histone modification patterns at genes involved in energy metabolism, oxidative stress responses, and inflammatory signaling [86].

Experimental and translational evidence suggests that altered maternal lipid exposure can modify histone acetylation at promoters and enhancers of genes regulating fatty acid oxidation and mitochondrial biogenesis in the placenta. Such modifications may initially represent adaptive responses that enhance placental metabolic flexibility and ATP production. However, sustained exposure to elevated FFAs and lipid-derived oxidative signals may disrupt the balance of histone modifications, leading to chromatin remodeling that impairs mitochondrial efficiency and energy homeostasis. These epigenetic changes may contribute to reduced placental metabolic capacity and altered nutrient delivery to the fetus, with potential implications for fetal growth and metabolic programming [87].

MicroRNAs (miRNAs) represent a third major epigenetic mechanism through which maternal lipid profiles influence placental and fetal gene regulation. These small non-coding RNAs modulate gene expression post-transcriptionally by binding to target mRNAs and regulating their stability or translation. Placental miRNA expression is highly responsive to metabolic and oxidative cues, including lipid availability and redox status. Several miRNAs implicated in lipid metabolism, mitochondrial function, and inflammatory pathways have been shown to respond to changes in maternal metabolic conditions, thereby influencing the expression of placental transporters and intracellular signaling networks [88].

Studies in humans have shown that placental miRNA profiles vary depending on maternal metabolic traits, including lipid levels and adiposity. These miRNAs target genes involved in fatty acid uptake, β-oxidation, and lipid storage. Although mechanistic details are still developing, these findings indicate that miRNA-mediated regulation plays a role in converting maternal lipid signals into coordinated changes in placental gene expression. By affecting key metabolic pathways, miRNAs may help regulate placental nutrient transfer and fetal lipid exposure during critical developmental periods [89].

Oxidative stress constitutes an important integrative mechanism linking maternal lipid metabolism to epigenetic regulation in the placenta. Increased flux of FFAs into placental mitochondria enhances β-oxidation and the generation of reducing equivalents, increasing electron input into the electron transport chain. While physiological levels of ROS generated under these conditions participate in normal placental signaling and vascular development, excessive or prolonged oxidative stress can disrupt redox-sensitive epigenetic processes. ROS can modulate the activity of DNA methyltransferases, histone-modifying enzymes, and miRNA expression, thereby coupling metabolic stress to long-lasting changes in gene regulation [90].

In human placental tissue, markers of oxidative stress have been linked to the altered expression of genes involved in lipid metabolism, antioxidant defense, and inflammatory signaling. These associations suggest that redox-dependent epigenetic mechanisms play a role in shaping placental responses to maternal lipid exposure and may influence fetal metabolic programming even in the absence of overt pathology. The interplay between lipid metabolism, mitochondrial function, and oxidative stress thus represents a central axis through which maternal metabolic status is translated into epigenetic information during pregnancy [91].

The integration of DNA methylation, histone modifications, and miRNA-mediated regulation forms a complex epigenetic network that enables the placenta and fetus to adapt to variations in maternal lipid availability. While this network supports physiological flexibility and resilience, it also renders the developing fetus sensitive to sustained metabolic perturbations [92]. Epigenetic alterations established in utero may persist into postnatal life, influencing mitochondrial function, insulin sensitivity, and lipid handling in metabolically active tissues. Observational studies have linked maternal lipid profiles and placental epigenetic markers with offspring adiposity and cardiometabolic risk indicators in childhood, highlighting the clinical relevance of these molecular mechanisms [93].

Human epigenome-wide association studies (EWASs) have provided evidence that maternal lipid profiles during pregnancy are associated with differential DNA methylation at defined genomic loci that are relevant for lipid and metabolic regulation in the fetus. For example, in the Healthy Start cohort, higher maternal triglycerides (TG) were associated with differential methylation at cord blood CpG sites annotated to ABCG1 (ATP binding cassette subfamily G member 1), a gene involved in cholesterol efflux and lipid transport, and methylation at these sites correlated with neonatal lipid traits and early adiposity measures [94]. Similarly, EWAS meta-analyses including multiple birth cohorts have repeatedly identified CpG sites near CPT1A (carnitine palmitoyltransferase 1A), a key enzyme for mitochondrial fatty acid β oxidation, where methylation levels associate with both maternal triglycerides and offspring lipid and glucose traits, highlighting a nexus between maternal lipid exposure, epigenetic regulation, and offspring metabolic phenotype [95]. Another site consistently reported in human cohorts is cg06500161 in ABCG1, whose methylation in cord blood correlates with maternal HDL cholesterol and TG levels and with child adiposity and insulin resistance later in life [96]. Beyond individual loci, pathway-level analyses show enrichment of CpG sites associated with maternal lipids in genes involved in lipid biosynthesis, PPAR and insulin signaling pathways, and oxidative phosphorylation, indicating that maternal lipid exposure is linked to coordinated epigenetic variation across metabolic gene networks echanistic interpretation connecting maternal lipids to offspring metabolic programming [97,98].

**Figure 3:**
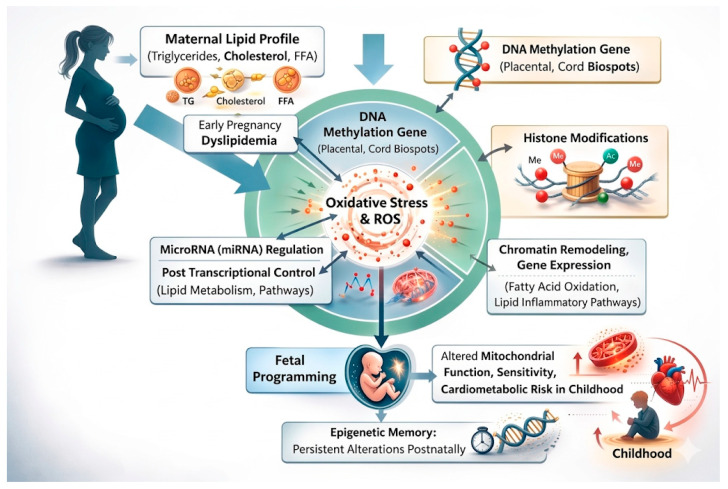
Mechanistic pathways of maternal lipid-induced epigenetic programming and offspring cardiometabolic risk.

In addition, maternal lipid-induced oxidative stress influences epigenetic remodeling in the placenta. Elevated free fatty acid flux increases mitochondrial reactive oxygen species (ROS), which can modulate DNA methyltransferase (DNMT) activity, histone acetyltransferase (HAT) function, and SIRT1-mediated histone deacetylation, contributing to altered regulation of genes involved in β-oxidation, antioxidant defense (e.g., SOD2, GPX1), and insulin signaling (IRS1, AKT2) [99]. Persistent epigenetic marks established in utero may therefore influence postnatal insulin sensitivity, hepatic lipid handling, adiposity distribution, and overall cardiometabolic risk, findings supported by longitudinal epigenome studies linking early-life epigenetic signatures with later metabolic outcomes The interplay among DNA methylation, histone modifications, microRNA regulation, and oxidative stress underscores the mechanistic link between maternal lipid metabolism and long-term offspring metabolic outcomes, reinforcing the developmental origins of health and disease (DOHaD) framework and providing potential targets for early nutritional and pharmacological interventions aimed at mitigating cardiometabolic risk from birth onward [100] (Figure 3).

During intrauterine life, maternal dyslipidemia (elevated triglycerides, cholesterol, and free fatty acids) triggers a cascade of epigenetic modifications, including DNA methylation (placental and cord blood samples), histone modifications (acetylation/methylation), and microRNA (miRNA) regulation. Central to this process is the generation of oxidative stress and reactive oxygen species (ROS), which act as mediators of chromatin remodeling and altered gene expression in fatty acid oxidation and lipid inflammatory pathways. These in utero changes establish an “epigenetic memory,” leading to fetal programming of altered mitochondrial function and sensitivity. Postnatally, these persistent alterations manifest as increased cardiometabolic risk in childhood, including predispositions to obesity, insulin resistance, and cardiovascular dysfunction.

## 8. Integrative Strategies for Maternal Metabolic Optimization and Fetal Programming

Maternal lipid metabolism, oxidative stress, inflammatory signaling, micronutrient status, and environmental exposures collectively modulate fetal growth, organ development, and long-term metabolic programming. Alterations in lipid metabolism during pregnancy affect placental function and epigenetic mechanisms in both the placenta and the fetus; maternal dyslipidemia has been associated with accelerated placental epigenetic aging, which may influence fetal growth and long-term offspring outcomes [101].

Dysregulated maternal lipid profiles, elevated reactive oxygen species (ROS), chronic inflammation, and micronutrient deficiencies contribute to oxidative stress and inflammatory signaling, thereby disrupting placental nutrient transport and impairing mitochondrial function. Oxidative stress occurs when ROS production exceeds antioxidant defense capacity, a condition frequently observed in pregnancies complicated by metabolic imbalance, including gestational diabetes and hyperlipidemia [102].

These disruptions may alter fetal epigenetic patterns, representing a mechanism through which intrauterine stress can exert long-term effects on offspring metabolism and health. Evidence indicates that oxidative stress and metabolic dysregulation can modify DNA methylation profiles, potentially increasing susceptibility to insulin resistance, dyslipidemia, neurodevelopmental impairment, and cardiometabolic disease later in life [103].

Longitudinal and mechanistic studies demonstrate that targeted dietary strategies, supplementation, lifestyle modification, and reduction in environmental stressors can modulate these pathways, thereby improving neonatal outcomes and supporting optimal fetal programming. Nutritional approaches aimed at optimizing lipid metabolism and antioxidant capacity—including balanced dietary patterns, adequate micronutrient intake, and minimization of pro-oxidant exposures—may mitigate these disruptions and promote healthy fetal development [104].

Maternal diet composition profoundly shapes lipid metabolism, oxidative balance, and fetal epigenetic landscape. The Mediterranean diet (MD), characterized by high intake of monounsaturated fatty acids (MUFAs, e.g., oleic acid), long-chain omega-3 polyunsaturated fatty acids (LC-PUFAs, e.g., DHA, EPA), polyphenols, carotenoids, fiber, and micronutrients (vitamins C, E, D, folate, B12, Fe, Mg, Zn), orchestrates multiple protective mechanisms [6]. MUFAs enhance phospholipid membrane fluidity, modulate nuclear receptor activity (PPARα/γ), reduce NF-κB-mediated inflammatory signaling, and limit lipid peroxidation. LC-PUFAs integrate into phospholipid membranes, improving placental lipid transport, suppressing pro-inflammatory eicosanoids, and activating pro-resolving mediators, thereby supporting fetal neurodevelopment and metabolic homeostasis [105,106].

Polyphenols, carotenoids, and flavonoids, abundant in olive oil, nuts, fruits, and vegetables, enhance endogenous antioxidant defense systems through activation of redox-sensitive transcription factors such as NRF2, upregulating enzymes including superoxide dismutase, catalase, and glutathione peroxidase, thereby attenuating ROS-induced lipid peroxidation, DNA damage, and mitochondrial dysfunction in placental and fetal tissues [107,108]. These compounds also modulate epigenetic regulators, including DNA methyltransferases (DNMTs) and histone deacetylases (HDACs), thereby influencing methylation and chromatin states of genes involved in lipid metabolism, insulin signaling, and adipogenesis [109]. Vitamin C, a water-soluble antioxidant, participates in enzymatic and non-enzymatic ROS scavenging, supports collagen biosynthesis, is important for placental vascular integrity, and recycles oxidized vitamin E, maintaining a synergistic redox network [110]. Vitamin E, a lipid-soluble antioxidant localized in cellular membranes, interrupts lipid peroxidation chain reactions, modulates NF κB-mediated inflammatory signaling, and preserves mitochondrial membrane integrity, contributing to maintenance of placental homeostasis during oxidative challenge [111].

Maternal dietary intake of polyphenol-rich foods has been associated with enhanced placental antioxidant status and modulation of DNA methylation patterns in experimental models of intrauterine growth restriction (IUGR), indicating potential for improved fetal metabolic programming [112]. Polyphenol supplementation combined with omega-3 fatty acids during pregnancy has demonstrated favorable effects on prenatal growth and metabolic biomarkers in animal models, suggesting synergistic benefits of combined antioxidant and lipid-modulating interventions [113]. Observational analyses and meta-analyses indicate that higher maternal intake of antioxidant vitamins, including vitamins C and E, correlates with reduced oxidative stress markers and improved fetal growth parameters, particularly in pregnancies complicated by metabolic imbalance or dyslipidemia [114]. Controlled trials of vitamin E supplementation in pregnancy have shown reductions in biomarkers of oxidative injury and inflammation in maternal and placental tissues, supporting the potential clinical relevance of targeted antioxidant interventions [115,116].

Vitamin D plays a multifaceted role in maternal–fetal physiology by modulating placental immune and metabolic pathways and influencing oxidative balance. Active vitamin D (1,25 dihydroxyvitamin D) downregulates pro-inflammatory cytokines such as interleukin 6 (IL 6) and tumor necrosis factor α (TNF α) while enhancing anti-inflammatory mediators, including IL 4 and IL 10, thereby contributing to attenuation of placental inflammation and oxidative stress [117,118]. In addition to its immunomodulatory effects, vitamin D influences enzymes involved in long-chain polyunsaturated fatty acid metabolism, including fatty acid desaturase isoforms (FADS1/2), which affects LC PUFA bioavailability and integration into placental lipid transport mechanisms that are essential for fetal neurodevelopment and metabolic homeostasis [119]. Beyond these genomic actions mediated through the vitamin D receptor (VDR), calcitriol has been implicated in epigenetic regulation of genes involved in lipid and glucose metabolism in trophoblast and fetal tissues, suggesting a potential role in fetal metabolic programming [120].

Observational studies consistently report that lower maternal serum 25-hydroxyvitamin D [25(OH)D] levels are associated with increased risk of preeclampsia, placental insufficiency, fetal growth restriction, and other adverse perinatal outcomes, implicating vitamin D insufficiency as a modifiable risk factor in pregnancy complications [121]. Systematic reviews further indicate that maternal vitamin D deficiency adversely affects placental function through dysregulation of angiogenic and nutrient transporter pathways, increases oxidative and inflammatory signaling within the placenta, and may contribute to impaired nutrient exchange and fetal growth [122]. Interventional studies demonstrate that maternal vitamin D supplementation can improve markers of placental function, reduce systemic inflammation, and support optimal fetal growth trajectories, although the precise dose–response relationships and optimal timing of supplementation require further clarification [123]. Adequate maternal vitamin D status reduces the risk of preeclampsia, placental insufficiency, and fetal growth restriction [124].

Iron (Fe) is essential for maternal oxygen transport, placental energy metabolism, and fetal growth. Pregnancy greatly increases iron requirements to support maternal red blood cell expansion and developing fetal and placental tissues, and both iron deficiency and iron excess have been linked to adverse outcomes. Maternal iron deficiency alters placental iron transport mechanisms and can reduce iron availability to the fetus, affecting placental function and fetal growth. Rodent models demonstrate that both low and high maternal iron intake disrupt placental iron handling and reproductive performance, while moderate iron supplementation improves fetal weight and reproductive outcomes, highlighting the importance of balanced iron status for placental physiology [125]. Human cohort data indicate that maternal iron supplementation is associated with increased offspring birth size and adiposity, potentially through effects on maternal metabolism and fetal growth trajectories, although long-term cardiometabolic consequences require further study [126]. Observational evidence also shows that iron deficiency in pregnancy impacts offspring neurodevelopment and child health outcomes, suggesting that maternal iron status contributes to long-term programming processes [127]. Optimal maternal iron status minimizes pro-oxidant effects of labile iron and supports controlled iron transport through placental mechanisms regulated by hepcidin and iron transport proteins, maintaining iron homeostasis in both mother and fetus [128].

Folate and vitamin B12 are central to one-carbon metabolism, providing methyl groups for S-adenosylmethionine synthesis, DNA methylation, and nucleotide biosynthesis [129]. Folate status in early pregnancy has been associated with placental growth and birth outcomes, with higher maternal serum folate correlating with improved outcomes, whereas folate deficiency is linked to reduced fetal growth parameters, reflecting the nutrient’s role in DNA synthesis and epigenetic regulation during critical developmental windows [130]. Although broad epigenome-wide evidence in humans remains limited, meta-analyses confirm that maternal vitamin B12 concentrations are associated with differential DNA methylation at multiple CpG sites in cord blood, with these epigenetic changes linked to birth outcomes such as birth weight and gestational age, underscoring one-carbon nutrients’ potential influence on fetal programming pathways [131].

Maternal protein intake, including essential amino acids such as arginine, methionine, and branched-chain amino acids (BCAAs), plays a crucial role in supporting placental perfusion, nitric oxide synthesis, and fetal growth. Arginine enhances endothelial nitric oxide synthase (eNOS) activity, improving uteroplacental blood flow and angiogenesis, while methionine participates in one-carbon metabolism alongside folate and vitamin B12, supporting DNA methylation and epigenetic programming. BCAAs contribute to maternal energy homeostasis and may mitigate oxidative stress by stabilizing maternal glucose and lipid flux [132]. Observational studies in large human cohorts indicate that maternal serum BCAA concentrations and protein intake are associated with neonatal anthropometric measures, including birth weight and fat mass, as well as growth trajectories extending into childhood [133]. Prospective cohort evidence further suggests that inadequate maternal protein intake correlates with increased risk of small-for-gestational-age (SGA) infants and alterations in postnatal BMI, highlighting the significance of adequate protein nutrition for fetal programming and early-life metabolic outcomes [134]. While interventional trials directly assessing protein or BCAA supplementation are limited, these findings underscore the importance of balanced maternal protein consumption within the context of overall dietary quality to support optimal perinatal outcomes [135].

Maternal physical activity during pregnancy has been associated with multiple beneficial effects on maternal metabolic health and perinatal outcomes. Systematic reviews of randomized controlled trials and observational cohorts indicate that moderate prenatal physical activity is linked to improved maternal glucose metabolism, reduced risk of gestational diabetes mellitus, better control of gestational weight gain, and lower incidence of hypertensive disorders of pregnancy, all of which indirectly support optimal placental function and fetal growth by enhancing nutrient utilization and reducing systemic metabolic stress [136,137]. Regular walking, aerobic, and strength-preserving exercise regimens have also been associated with better maternal cardiovascular profiles and may contribute to healthier neonatal anthropometry, although the magnitude of effects on direct fetal programming endpoints requires further investigation [138,139].

In contrast, maternal exposure to chronic psychological stress is linked to adverse perinatal outcomes through activation of the maternal hypothalamic pituitary adrenal (HPA) axis and downstream stress signaling pathways. Elevated maternal cortisol and associated stress biomarkers have been associated with altered placental gene expression and epigenetic modifications, including differential DNA methylation patterns in placental and cord blood tissues, and with increased risks of preterm birth, small—gestational-age (SGA) neonates, and neurodevelopmental differences in offspring [140]. Large human cohort studies have identified associations between maternal perceived stress during gestation and epigenetic changes in offspring at glucocorticoid-related loci and stress-responsive pathways, supporting the role of prenatal stress in fetal programming of metabolic and neuroendocrine systems [141].

Together, these data support the inclusion of structured moderate physical activity and psychosocial stress mitigation strategies in comprehensive prenatal care, as components that may synergize with nutritional optimization to reduce oxidative and metabolic stress, promote placental efficiency, and favor more adaptive fetal developmental trajectories [142].

Physical activity synergistically reinforces dietary effects. Moderate exercise enhances mitochondrial biogenesis, insulin sensitivity, antioxidant enzyme activity, and systemic anti-inflammatory status. It activates redox-sensitive transcription factors (NRF2, PGC-1α), optimizes placental vascularization and nutrient sensing, and supports fetal epigenetic regulation. Regular exercise modulates ROS, stabilizes redox-sensitive signaling pathways, and improves maternal lipid profiles, collectively promoting fetal metabolic programming [143,144].

Maternal exposure to environmental pollutants, including airborne particulate matter (PM_2.5_, PM_10_, NO_2_), phthalates (esters of phthalic acid commonly used as plasticizers in consumer products such as plastics, cosmetics, and food packaging), and industrial chemicals, has been associated with significant effects on placental function and fetal development. Prenatal exposure to PM and other air pollutants induces oxidative stress and inflammatory responses in placental tissues, which can alter DNA methylation patterns and gene expression of pathways involved in angiogenesis, nutrient transport, and fetal growth regulation [145,146]. Epigenome-wide association studies in human cohorts have shown that maternal exposure to phthalates and air pollutants correlates with differential DNA methylation in cord blood and placenta, suggesting programming effects on offspring metabolic and neurodevelopmental pathways [147].

Mechanistically, these pollutants increase ROS production in trophoblast cells, activate inflammatory signaling cascades, and modulate one-carbon metabolism and methylation potential, contributing to aberrant epigenetic signatures in the developing placenta. Altered epigenetic patterns have been linked to adverse perinatal outcomes, including reduced birth weight, small-for-gestational-age (SGA) neonates, and increased susceptibility to metabolic and cardiovascular disorders later in life. The timing of exposure is critical, with early gestational exposure often producing more pronounced epigenetic effects due to the heightened sensitivity of placental developmental processes during this window [148].

Together, these findings underscore the importance of mitigating maternal exposure to environmental stressors as part of comprehensive prenatal care. Reducing exposure to air pollution, industrial chemicals, and endocrine-disrupting compounds such as phthalates may help preserve placental epigenetic integrity, optimize nutrient transfer, and promote healthier fetal growth trajectories, complementing nutritional and lifestyle interventions for perinatal programming [149].

Targeted supplementation within a Mediterranean-based framework enhances maternal–fetal outcomes. LC-PUFAs reduce pro-inflammatory eicosanoids and stimulate pro-resolving mediators; vitamins C and E mitigate lipid peroxidation and restore redox-sensitive pathways; folate and vitamin B12 maintain DNA methylation and histone modifications, regulating genes for lipid metabolism, insulin signaling, and adipogenesis; vitamin D and Fe optimize perfusion, antioxidant defenses, and epigenetic regulation. Dosing and timing must be individualized according to maternal lab parameters to maximize efficacy while avoiding potential toxicity [150,151].

Preconception optimization—including Mediterranean diet adherence, micronutrient sufficiency, and regular moderate physical activity—reduces the likelihood of dyslipidemia, oxidative stress, and inflammation during pregnancy. Early prenatal education regarding nutrient-rich diets, micronutrient adequacy, and avoidance of pro-oxidants enhances continuity of care from preconception through postpartum, improving placental function, fetal growth, and long-term metabolic health [152,153].

**Figure 4:**
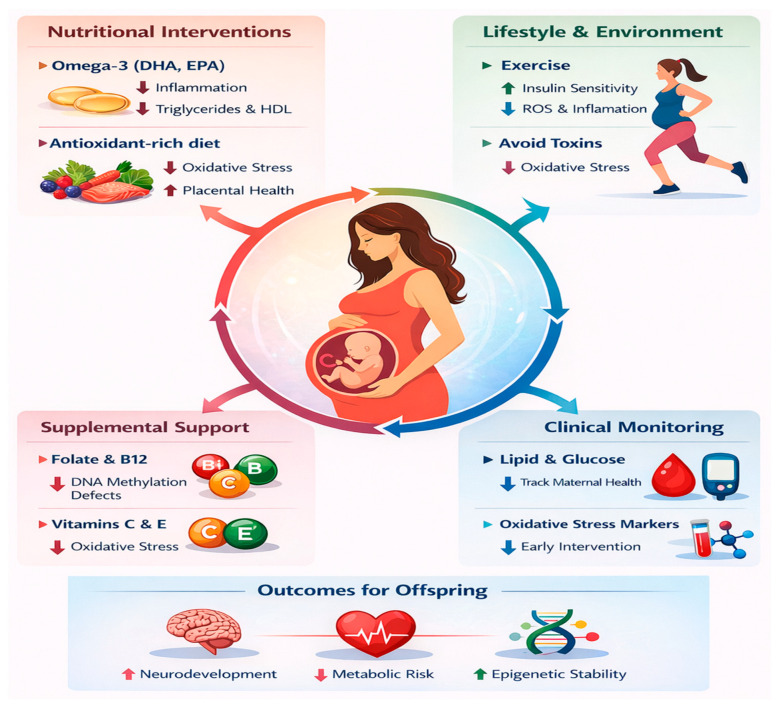
Integrative maternal-fetal metabolic optimization and epigenetic programming. Integrative maternal strategies represent a multimodal framework for the optimization of the fetal metabolic milieu through the precise modulation of nutritional, pharmacological, and behavioral determinants. The synergistic effect of a low-glycemic dietary regimen, a reduction in saturated lipid intake, and targeted supplementation with omega-3 polyunsaturated fatty acids (DHA/EPA) directly correlates with the attenuation of maternal systemic inflammation and the suppression of pro-oxidative processes. These factors, alongside an adequate intake of methyl donors such as folate and vitamin B12, stabilize redox-sensitive signaling pathways and prevent the activation of pro-inflammatory cascades (such as the NF-κB pathway) within the placental barrier.

Clinical surveillance of maternal metabolism—systematic evaluation of lipid panels, blood glucose, oxidative stress indices, inflammatory markers, and micronutrient status—allows for prompt, individualized adjustments in diet, physical activity, and targeted supplementation. The combination of laboratory-guided assessments with personalized counseling facilitates adaptive maternal metabolic management, supporting fetal growth and development while enhancing the efficacy of a Mediterranean-style dietary pattern and other health-promoting behaviors [154] (Figure 4).

## 9. Conclusions

Monitoring maternal lipid profiles, nutritional status, physical activity, psychosocial stress, and exposure to environmental pollutants is crucial for optimizing the intrauterine environment and promoting fetal health. Dysregulated maternal metabolism, oxidative stress, and adverse exposures can impair placental function, compromise nutrient transfer, and induce epigenetic modifications in the fetus, contributing to intergenerational transmission of metabolic risk. Individualized approaches—including adherence to a Mediterranean diet, micronutrient supplementation tailored to laboratory parameters, moderate prenatal physical activity, stress mitigation strategies, and minimizing pollutant exposure—collectively support placental efficiency, fetal growth, and long-term offspring health.

Future research should focus on elucidating the precise molecular mechanisms by which lifestyle and environmental factors interact with placental epigenetic programming, defining the optimal timing and dosage of dietary and supplementation interventions, and translating these findings into personalized prenatal care strategies that integrate nutrition, lifestyle, and environmental risk management.

## Data Availability

No new data were created or analyzed in this study. Data sharing is not applicable to this article.
